# Benefits and challenges in implementation of artificial intelligence in colonoscopy: World Endoscopy Organization position statement

**DOI:** 10.1111/den.14531

**Published:** 2023-03-13

**Authors:** Yuichi Mori, James E. East, Cesare Hassan, Natalie Halvorsen, Tyler M. Berzin, Michael Byrne, Daniel von Renteln, David G. Hewett, Alessandro Repici, Mohan Ramchandani, Maryam Al Khatry, Shin‐ei Kudo, Pu Wang, Honggang Yu, Yutaka Saito, Masashi Misawa, Sravanthi Parasa, Carolina Ogawa Matsubayashi, Haruhiko Ogata, Hisao Tajiri, Nonthalee Pausawasdi, Evelien Dekker, Omer F. Ahmad, Prateek Sharma, Douglas K. Rex

**Affiliations:** ^1^ Clinical Effectiveness Research Group University of Oslo Oslo Norway; ^2^ Department of Transplantation Medicine Oslo University Hospital Oslo Norway; ^3^ Digestive Disease Center Showa University Northern Yokohama Hospital Kanagawa Japan; ^4^ Endoscopy Division National Cancer Center Hospital Tokyo Japan; ^5^ Center for Diagnostic and Therapeutic Endoscopy, School of Medicine Keio University Tokyo Japan; ^6^ Jikei University School of Medicine Tokyo Japan; ^7^ Translational Gastroenterology Unit, John Radcliffe Hospital University of Oxford Oxford UK; ^8^ NIHR Oxford Biomedical Research Centre Oxford UK; ^9^ Division of Gastroenterology and Hepatology Mayo Clinic Healthcare London UK; ^10^ University College London London UK; ^11^ Department of Biomedical Sciences Humanitas University Pieve Emanuele Italy; ^12^ Endoscopy Unit Humanitas Clinical and Research Center ‐ IRCCS Rozzano Italy; ^13^ Division of Gastroenterology Beth Israel Deaconess Medical Center and Harvard Medical School Boston USA; ^14^ Swedish Medical Center Seattle USA; ^15^ Division of Gastroenterology and Hepatology University of Kansas School of Medicine and VA Medical Center Kansas City USA; ^16^ Division of Gastroenterology Indiana University School of Medicine Indianapolis USA; ^17^ Department of Medicine The University of British Columbia Vancouver Canada; ^18^ Division of Gastroenterology University of Montreal Medical Center (CHUM) and Research Center (CRCHUM) Montreal Canada; ^19^ School of Medicine The University of Queensland Brisbane Australia; ^20^ Asian Institute of Gastroenterology Hyderabad India; ^21^ Department of Gastroenterology Obaidulla Hospital Ras Al Khaimah United Arab Emirates; ^22^ Sichuan Academy of Medical Sciences and Sichuan Provincial People's Hospital Chengdu China; ^23^ Department of Gastroenterology Renmin Hospital of Wuhan University Wuhan China; ^24^ Gastrointestinal Endoscopy Unit, Gastroenterology Department University of São Paulo Medical School São Paulo Brazil; ^25^ Vikit Viranuvatti Siriraj GI Endoscopy Center Mahidol University Bangkok Thailand; ^26^ Division of Gastroenterology, Department of Medicine, Faculty of Medicine, Siriraj Hospital Mahidol University Bangkok Thailand; ^27^ Department of Gastroenterology and Hepatology Amsterdam University Medical Center Amsterdam The Netherlands

**Keywords:** colon polyp, colonoscopy

## Abstract

The number of artificial intelligence (AI) tools for colonoscopy on the market is increasing with supporting clinical evidence. Nevertheless, their implementation is not going smoothly for a variety of reasons, including lack of data on clinical benefits and cost‐effectiveness, lack of trustworthy guidelines, uncertain indications, and cost for implementation. To address this issue and better guide practitioners, the World Endoscopy Organization (WEO) has provided its perspective about the status of AI in colonoscopy as the position statement. **WEO Position Statement**: Statement 1.1: Computer‐aided detection (CADe) for colorectal polyps is likely to improve colonoscopy effectiveness by reducing adenoma miss rates and thus increase adenoma detection; Statement 1.2: In the short term, use of CADe is likely to increase health‐care costs by detecting more adenomas; Statement 1.3: In the long term, the increased cost by CADe could be balanced by savings in costs related to cancer treatment (surgery, chemotherapy, palliative care) due to CADe‐related cancer prevention; Statement 1.4: Health‐care delivery systems and authorities should evaluate the cost‐effectiveness of CADe to support its use in clinical practice; Statement 2.1: Computer‐aided diagnosis (CADx) for diminutive polyps (≤5 mm), when it has sufficient accuracy, is expected to reduce health‐care costs by reducing polypectomies, pathological examinations, or both; Statement 2.2: Health‐care delivery systems and authorities should evaluate the cost‐effectiveness of CADx to support its use in clinical practice; Statement 3: We recommend that a broad range of high‐quality cost‐effectiveness research should be undertaken to understand whether AI implementation benefits populations and societies in different health‐care systems.

## INTRODUCTION

**Figure 1:**
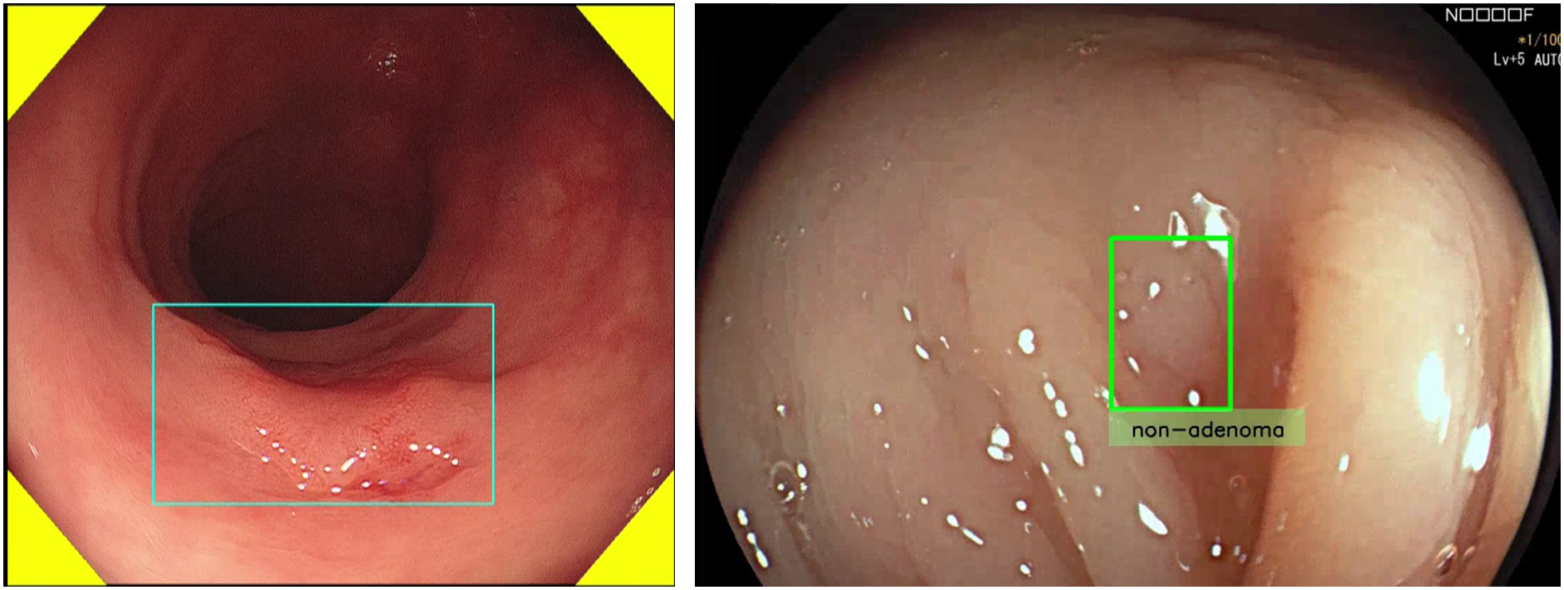
Left, computer‐aided detection for colorectal polyps (EndoBRAIN; Olympus Corp., Tokyo, Japan); right, computer‐aided diagnosis for colorectal polyps (GI GENIUS CADx; Medtronic Corp., Dublin, Ireland).

Application of artificial intelligence (AI) has gained significant attention as a novel measure to allow standardization in colonoscopy practice. Computer‐aided detection (CADe) and computer‐aided diagnosis (CADx) are the major uses for AI technologies, aiming to help endoscopists detect and characterize polyps during colonoscopy (Fig. 1). More than 10 high‐quality, randomized controlled trials (RCTs) have been published in the past couple of years, and 10 or more CADe and CADx medical devices are now commercially available on the global market.^1^, ^2^

Nevertheless, these AI medical devices have not been widely implemented in clinical endoscopy practice.^2^ The major reasons may include lack of data on clinical benefits and cost‐effectiveness, lack of trustworthy guidelines, uncertain indications and training requirements, cost for implementation, and probably lack of reimbursement. Furthermore, lack of adequate communication between scientific communities and health‐care delivery systems might also hinder the implementation process of these AI tools. Because a variety of issues hinder the implementation process, it becomes difficult for practitioners to understand whether to incorporate these novel tools into routine colonoscopy practice. To better guide practitioners, we developed a position statement project focused on AI in colonoscopy with a clear perspective from the World Endoscopy Organization (WEO). This statement also emphasizes the importance of engaging with health‐care delivery systems and authorities, because these entities usually play important roles in the implementation process of innovative medical devices.

## METHODS

This position statement is the output of an expert working group of the WEO Colorectal Cancer Screening Committee. The recommendations were developed based on a modified Delphi process.^3^ Topics for the review were initially developed by the steering committee members who were gastroenterologists with expertise in AI for colonoscopy (Appendix S1). These topics include major clinical benefits and challenges that can either facilitate or hinder clinical implementation of AI in colonoscopy practice.

The steering committee invited a total of 18 panel members (Appendix S1). They were selected in consideration of diversity in gender (6 female members and 12 male) and geography (4 from North America, 3 from Europe, 1 from Oceania, 8 from Asia, 1 from Latin America, and 1 from the Middle East). All of them were considered to have expertise in gastroenterology and AI according to their publication records.

Based on the initial topics proposed by the steering committee and the feedback from the panel members in a teleconference, structured statements were developed in March 2022. These statements consisted of three different categories: (i) CADe; (ii) CADx; and (iii) promotion of research. The panel members were asked to indicate their perspectives with each statement by using a Likert scale with five possible answers (strongly agree, agree, neutral, disagree, strongly disagree). They were also asked to make comments in a free‐text box per each statement. Consensus was confirmed when 80% or more chose either “strongly agree” or “agree”. Statements that did not reach the consensus threshold during the voting processes were either removed or modified in accordance with discussion after each voting process. The voting processes were done anonymously. There were three voting rounds in total between April and September 2022. Inclusion of 18 panel members and 83% (15/18) response rate in the final voting satisfied minimal standard required for the Delphi survey study.^4^, ^5^, ^6^ The final version of the developed statements was reviewed and approved by the WEO leadership on 10 November 2022. The statements were also endorsed by the Japan Gastroenterological Endoscopy Society on 28 November 2022.

## RECOMMENDATIONS

There were three statements which were evaluated in the first‐round voting (Appendix S2). After the first‐round voting and subsequent discussion with the panel members, the steering committee decided to split these statements into seven distinct and detailed statements. This revision was done because the committee considered that the first draft statements were too comprehensive and included different types of information within each of the statements. The seven developed statements were then assessed in the second‐round voting (Appendix S3). After this round, the steering committee further split one statement into two and modified wording of several statements in accordance with the discussion with the panel members. Thereafter, a total of eight statements were assessed in the third‐round voting in which 15 out of the 18 panel members participated (Appendix S4). Finally, seven statements out of the eight reached consensus level and were included in the final version as follows.

### Computer‐aided detection

*Statement 1.1*: CADe for colorectal polyps is likely to improve colonoscopy effectiveness by reducing adenoma miss rates and thus increase adenoma detection. (100% agreement).
*Statement 1.2*: In the short term, use of CADe is likely to increase health‐care costs by detecting more adenomas. (80% agreement).
*Statement 1.3*: In the long term, the increased cost by CADe could be balanced by savings in costs related to cancer treatment (surgery, chemotherapy, palliative care) due to CADe‐related cancer prevention. (80% agreement).
*Statement 1.4*: Health‐care delivery systems and authorities should evaluate the cost‐effectiveness of CADe to support its use in clinical practice. (100% agreement).

Missing neoplastic lesions is one of the biggest challenges in colonoscopy, which is considered a major cause of postcolonoscopy colorectal cancer. A recent meta‐analysis suggested 26% of adenomas are missed during colonoscopy.^7^ While some of the missed adenomas were overlooked due to the limited exposure of mucosal surface,^8^ an image‐based retrospective study suggested 14% of adenomas were not recognized by endoscopists even though they were visualized.^9^ CADe has been developed to support endoscopists by indicating the location of possible polyps on the monitor (Fig. 1). This may help reduce the number of missed adenomas^10^, ^11^ and thus increase detection rate of adenomas (ADR). Indeed, a recent meta‐analysis of six RCTs including 4354 patients demonstrated a relative increase of ADR of 44% with CADe.^12^ In this regard, CADe offers promise to reduce unwanted operator‐dependent variability in colonoscopy performance and thus maximize colonoscopy effectiveness.^13^ However, we should also take into account that CADe cannot pick up any adenomas that are not visualized. Addition of mucosal exposure devices may contribute to further detection of adenomas.^14^

**Figure 2:**
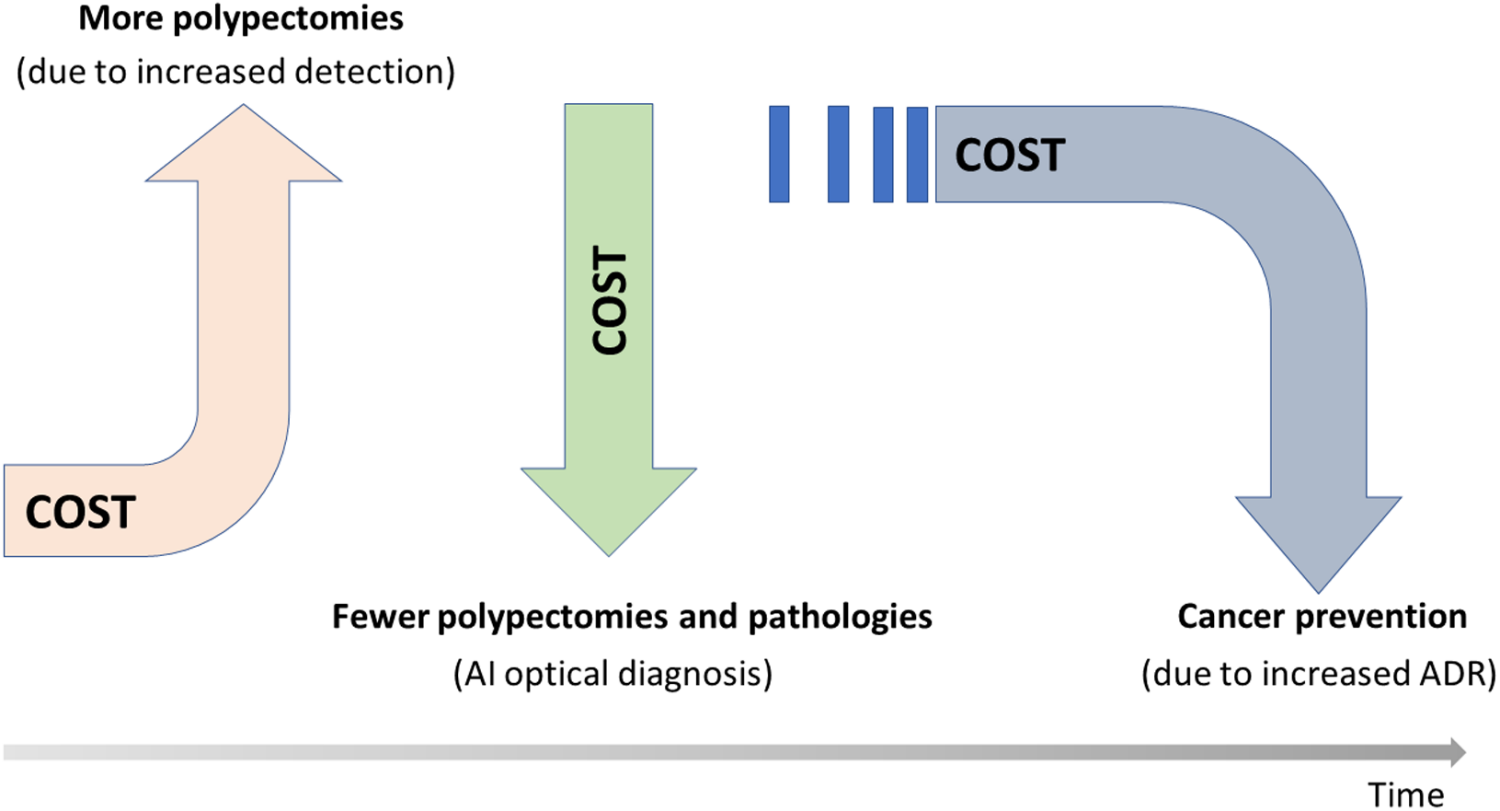
Possible cost increase and reduction introduced by artificial intelligence (AI) in colonoscopy. ADR, adenoma detection rate.

While clinical effectiveness is widely considered to be the most important factor for medical innovation, health economic evaluation is another critical area to be considered for implementation in clinical practice. Most health‐care systems undertake health technology assessments to inform decisions regarding the adoption and reimbursement of new innovations, with increasing emphasis placed on cost‐effectiveness given the trend to use scarce resources more efficiently within constrained budgets. A recent microsimulation study suggested that the use of CADe can increase health‐care cost in the short term by increasing the number of detected polyps, polypectomies, and histopathological examinations, in addition to the cost of CADe^15^ (Fig. 2). Furthermore, another simulation study suggested the number of intensive surveillance colonoscopies (e.g., 3‐year follow‐up) may increase by 35% in the United States,^16^ which is another burden that needs to be addressed.

On the contrary, CADe‐driven increases in ADR may contribute to reduction of colorectal cancer in the long term, which may lead to cost reduction. The same microsimulation study suggested the use of CADe could contribute to an absolute 5% and 3% reduction of colorectal cancer incidence and death, respectively, when compared to standard colonoscopy‐based screening in the United States. This reduction of colorectal cancer could result in savings of overall health‐care costs by roughly $290 million annually in the United States^15^ (Fig. 2). In this regard, health‐care delivery systems and authorities should evaluate the cost‐effectiveness of CADe to support its use in clinical practice.

However, there may be two major hurdles that hinder translation of this data into the real‐world health‐care arena. First, analyses are limited in terms of methodologies and geographical diversity. For now, cost‐effectiveness was evaluated in only one study considering the US health‐care system. Second, there are always significant uncertainties in the microsimulation modeling due to many assumptions. Large‐scale observational studies and/or RCTs with long‐term follow‐up will be required to understand if the use of CADe truly contributes to health economy and patient care.^1^

In addition to the cost‐related issues it is quite important to evaluate and discuss possible burdens and harms that CADe may bring to clinical practice. This may include longer procedure time due to the prolonged withdrawal time and increased polypectomies,^17^ and psychological distraction of the endoscopists due to many false positive signals on the monitor.^18^ Furthermore, the benefits and harms induced by CADe may vary according to the expertise of endoscopists,^19^ which should be taken care of to achieve efficient introduction of this new technology.

### Computer‐aided diagnosis

*Statement 2.1*: CADx for diminutive polyps (≤5 mm), when it has sufficient accuracy, is expected to reduce health‐care costs by reducing polypectomies, pathological examinations, or both. (93.3% agreement).
*Statement 2.2*: Health‐care delivery systems and authorities should evaluate the cost‐effectiveness of CADx to support its use in clinical practice. (92.9% agreement).

Polyp characterization, or optical diagnosis, is a method to predict histopathology of a polyp based on its appearance. This can be used as an alternative to actual histopathological assessment when it is predicted with high confidence.^20^ The concept of optical diagnosis has been gaining significant attention because it can help select appropriate treatment measures in accordance with the predicted histopathology (e.g., no treatment for a hyperplastic polyp, endoscopic treatment for an adenoma, and surgery for a cancer), without a need to take pretreatment biopsy. This will reduce costs and patients' burden as long as prediction provides sufficient accuracy.^20^, ^21^ However, the lack of endoscopists' competence and motivation in polyp characterization has hindered implementation of optical diagnosis in clinical practice.^22^, ^23^ A large‐scale clinical trial conducted in community‐based hospitals in the UK showed that the sensitivity and specificity to identify adenomas were limited to 83% and 74%, respectively.^22^ Furthermore, a nation‐wide questionnaire survey in the United States revealed that only 40% of US endoscopists are willing to perform optical diagnosis.^23^

Use of CADx is expected to play a significant role to overcome these challenges because it can reduce the operator‐dependent uncertainties in optical diagnosis and assure prediction accuracy. The main arena where CADx contributes to real‐world colonoscopy for now is differentiation between neoplasia and non‐neoplasia, although several preclinical, advanced CADx are being investigated.^24^ Several large‐scale prospective studies have demonstrated the great value of using CADx to correctly differentiate diminutive polyps with over 90% negative predictive value and over 80% sensitivities/specificities for identification of adenomas,^25^, ^26^ which exceeded the threshold required for optical diagnosis in the United States^20^ and Europe.^21^

This indicates that introduction of CADx‐driven optical diagnosis can lead to significant reduction of polypectomy and pathology‐related cost in colonoscopy.^27^ A post‐hoc analysis of a large‐scale prospective study showed the use of CADx could lead to 11% reduction of average colonoscopy cost with at most $85.2 million saving in the United States.^27^ In this regard, health‐care delivery systems and authorities should evaluate the cost‐effectiveness of CADx to support its use in clinical practice. Furthermore, the importance of CADx is further emphasized with the broader use of CADe that may increase detection of small polyps, many of which indeed are hyperplastic polyps.^12^ CADx may play an important role to minimize the number of polypectomies which CADe increases (Fig. 2).

On the other hand, there have been several challenges that hinder clinical implementation of CADx. First, the additional value of using CADx to optical diagnosis is uncertain.^28^, ^29^ Two large‐scale prospective studies showed use of CADx did not increase the sensitivity to identify adenomas when compared with standard optical diagnosis.^28^, ^29^ However, the introduction of CADx obviously increased the proportion of high confidence diagnosis (74–93%)^29^ and motivation to adopt optical biopsy (40–57%)^23^ which will lead to increased use of optical diagnosis. Second, the benefits of CADx may be limited to nonexperienced endoscopists.^28^ Third, there is a relative lack of both number and variety of health economic studies. For now, we have a couple of simulation results in the United States, the UK, Norway, and Japan based on the leave‐in‐situ strategy for rectosigmoid polyps,^20^ while there is no other data supporting the broader use of CADx from an economic point of view, including the lack of data focused on resect‐and‐discard strategies for a whole colon.^20^ Fourth, clinical implementation of CADx, compared to CADe, faces resistance among practitioners related to perceived medical‐legal risk that is particularly associated with the resect‐and‐discard strategy. Practices and policies that reduce risk, such as systematic photo documentation or videorecording of endoscopic features that support diagnoses and clinical management decisions, combined with financial incentives to physicians who utilize CADx, may be essential to the effective spread of CADx in clinical practice.

### Promotion of research

*Statement 3*: We recommend that a broad range of high‐quality cost‐effectiveness research should be undertaken to understand whether AI implementation benefits populations and societies in different health‐care systems. (100% agreement).

In contrast to the rapid pace of development and translation for AI based technologies in health care, cost‐effectiveness research has dramatically lagged behind the speed of technological innovation. A recent systematic review identified only 20 health economic studies focused on AI medicine.^30^ To date, only two cost‐effectiveness studies for AI in colonoscopy have been published, relating to CADx^27^ and CADe.^15^ Considering the potential global applications for AI in colonoscopy, it is critical that future research addresses cost‐effectiveness in different health‐care systems internationally. This is simply because there are huge variations in health‐care delivery systems, socioeconomic status, and policies of reinbursement (e.g., incentivizing outcomes instead of volume, utilizing advance market commitments, and time‐limited reimbursements^31^).

In addition, more robust health economic evaluation will depend upon long‐term clinical outcome data, such as the impact on colorectal cancer incidence, as opposed to simulation‐based modeling which is limited by assumptions. Furthermore, more complex economic evaluation will also be required given the fact that endoscopists are likely to use multiple AI tools in their future practice. This may include simultaneous use of both CADe and CADx as part of multialgorithm endoscopic “full workflow solutions” for colonoscopy procedures.

## DISCUSSION

We have reached a consensus about the importance of addressing both clinical benefits and cost‐effectiveness in relation to the implementation of both CADe and CADx in colonoscopy. However, implementation of a new innovative medical device in clinical practice is a huge challenge because there are multiple factors that affect this process, including regulatory approval, indications, clinical benefits, requirement of training, cost‐effectiveness, cost for implementation, reimbursement, physicians' sentiment, and legal and ethical challenges. In this regard, some of the optimistic results in the recent clinical studies in the AI colonoscopy field are just part of the process.

Many consider financial support from health insurance bodies, such as reimbursement, to be the strongest driving force in implementation. In fact, this is well illustrated by the use of AI in mammography. The US Food and Drug Administration approved CADe for mammography in 1998, and Medicare and Medicaid have reimbursed its use since 2002. As a result, AI detection tools are used for more than 80% of the screening mammograms in the United States.^32^ However, introduction of reimbursement is not straightforward. Recently, experts in health economy cautioned that per‐use reimbursement may result in the overuse of AI, and thus careful design of payment for AI is essential for improving patient outcomes while maximizing cost‐effectiveness and equity.^31^

Given that multidisciplinary issues potentially hinder smooth implementation, we strongly encourage health‐care delivery systems and authorities to consider multiple factors including the clinical benefits, cost‐effectiveness, or other formal health technology assessment of AI tools to support use in clinical practice.

## CONFLICT OF INTEREST

Y.M.: Olympus Corporation (speaker fee, consultancy and equipment on loan) and Cybernet Systems Corporation (ownership interest). J.E.E.: Satisfai Health (ownership interest and consultancy); Medtronic, Falk and Jannsen (speaker fee); PAION (consultancy). C.H.: Medtronic (consultancy and equipment on loan), Fujifilm (consultancy and equipment on loan) and Pentax (consultancy). O.F.A.: Olympus Corporation (speaker fee). T.M.B.: Medtronic, Wision A.I., Magentiq Eye, DocBot and RSIP Vision (consultancy). M.B.: Satisfai Health (CEO and shareholder). D.v.R.: ERBE, Ventage, Pendopharm, Fujifilm and Pentax (research funding); Boston Scientific, ERBE, Fujifilm and Pendopharm (consultancy). D.G.H.: Olympus Australia Pty Ltd and Fresenius Kabi Pty Ltd (consulting fee); Olympus Australia Pty Ltd, Fresenius Kabi Pty Ltd and Boston Scientific Pty Ltd (speaker honorarium). A.R.: Medtronic and Fujifilm (consultancy and equipment on loan). S.K.: Olympus Corporation (speaker fee) and Cybernet Systems Corporation (ownership interest). M.M.: Olympus Corporation (speaker fee and consultancy), Cybernet Systems Corporation (ownership interest) and Associate Editor of *Digestive Endoscopy*. S.P.: Covidien LP, Fujifilm USA, Mahana Therapeutics, Allen Institute for Artificial Intelligence and Quasal AI (consultancy); Fujifilm USA and Paul Allen Center for Cancer Research (grant support). C.O.M.: AI Medical Service Inc (employee). H.O.: AbbVie GK, Mochida Pharmaceutical Co., Ltd., Kyorin Pharmaceutical Company, Limited, Mitsubishi Tanabe Pharma Corporation, Otsuka Pharmaceutical Co., Ltd, Takeda Pharmaceutical Company Limited, EA Pharma Co., Ltd, JIMRO Co., Ltd, ZERIA Pharmaceutical Co., Ltd and Boston Scientific Corporation (donation towards scholarships); Olympus Corporation and Takeda Pharmaceutical Company Limited (consulting fee); Takeda Pharmaceutical Company Limited, Janssen Pharmaceutical K.K., Mitsubishi Tanabe Pharma Corporation, Mochida Seiyaku Co., Ltd, EA Pharma Co., Ltd, Covidien Japan Inc., AbbVie GK, Kyorin Pharmaceutical Company, Limited, Pfizer Inc., Otsuka Pharmaceutical Co., Ltd, Fujifilm Medical Co., Ltd, Olympus Corporation and Viatris Inc. (speaker fee). E.D.: Fujifilm (device on loan, consultancy, research grant and speaker fee); Olympus, GI Supply, PAION and Ambu (consultancy); Olympus, GI Supply, Norgine, IPSEN and PAION (speaker fee). P.S.: Medtronic, Olympus Corporation, Boston Scientific, Fujifilm, Salix Pharmaceuticals and Lumendi (consultancy); Ironwood Pharmaceuticals, Erbe, Docbot, Cosmo Pharmaceuticals and CDx Laboratories, Inc. (research grant). D.K.R.: Olympus Corporation, Boston Scientific, Aries Pharmaceutical, Braintree Laboratories, Lumendi, Norgine, Endokey, GI Supply, Medtronic and Acacia Pharmaceuticals (consultancy); Olympus Corporation, Medivators, Erbe USA Inc and Braintree Laboratories (research support); Satisfai Health (shareholder). The other authors declare no conflict of interest for this article.

## FUNDING INFORMATION

Author Y.M. is funded by the European Commission (Horizon Europe 101057099) and Japan Society for Promotion of Science (22H03357); J.E.E. is funded by the National Institute for Health Research (NIHR) Oxford Biomedical Research Centre. The views expressed are those of the authors and not necessarily those of the National Health Service, the NIHR, or the Department of Health.

## Supporting information


**Appendix S1.** Steering committee and panel members.

**Appendix S2.** Results of the first voting.

**Appendix S3.** Results of the second voting.

**Appendix S4.** Results of the third voting.
