# Supplementary material for: Benefits and challenges in implementation of artificial intelligence in colonoscopy: World Endoscopy Organization position statement
Source: Dig Endosc. 2023 Mar 13;35(4):422–9. doi: 10.1111/den.14531 (PMC12136278; doi:10.1111/den.14531)
Supplement: Supplementary file 1 — Appendix S1. Steering committee and panel members. [file DEN-35-422-s003.docx]

**Supplementary material 1: Steering committee and panel members**

**Steering committee:** Yuichi Mori, James East, Cesare Hassan, Omer F Ahmad, Prateek Sharma, and Douglas K Rex

**Panel members:** Natalie Halvorsen, Tyler M. Berzin, Michael Byrne, Daniel von Renteln, David Hewett, Alessandro Repici, Mohan Ramchandani, Maryam Al Khatry, Wang Pu, Honggang Yu, Yutaka Saito, Masashi Misawa, Sravanthi Parasa, Carolina Ogawa Matsubayashi, Haruhiko Ogata, Hisao Tajiri, Nonthalee Pausawasdi, Evelien Dekker
