# Supplementary material for: Benefits and challenges in implementation of artificial intelligence in colonoscopy: World Endoscopy Organization position statement
Source: Dig Endosc. 2023 Mar 13;35(4):422–9. doi: 10.1111/den.14531 (PMC12136278; doi:10.1111/den.14531)
Supplement: Supplementary file 2 — Appendix S2. Results of the first voting. [file DEN-35-422-s001.docx]

**Supplementary material 2: Results of the 1^st^ voting**

***1. Computer-aided detection (CADe)***

Computer-aided detection (CADe) is likely to increase health care costs by detecting more adenomas in the short term while improving the quality of colonoscopy at the same time. In the long-term, this cost increment could be balanced by the impact of improved cancer prevention due to the increased detection and resection of adenomas. Health insurance bodies may consider the introduction of reimbursement if reliable data exist for the cost-effectiveness of CADe. (74% agreement)

***2. Computer-aided diagnosis (CADx)***

Computer-aided diagnosis (CADx) is likely to reduce health care costs by reducing unnecessary polypectomies of diminutive polyps (<=5mm) and pathological examinations. Health insurance bodies should consider the introduction of reimbursement if reliable cost-effectiveness data exist for CADx. (100% agreement)

***3. Promotion of research***

We recommend that a greater variety of high-quality cost-effectiveness research should be undertaken to understand if AI-implementation benefits populations and societies in different health care systems. (95% agreement)
