# Supplementary material for: Benefits and challenges in implementation of artificial intelligence in colonoscopy: World Endoscopy Organization position statement
Source: Dig Endosc. 2023 Mar 13;35(4):422–9. doi: 10.1111/den.14531 (PMC12136278; doi:10.1111/den.14531)
Supplement: Supplementary file 3 — Appendix S3. Results of the second voting. [file DEN-35-422-s004.docx]

**Supplementary material 3: Results of the 2^nd^ voting**

***1. Computer-aided detection (CADe)***

1.1 Computer-aided detection (CADe) is likely to improve colonoscopy effectiveness by reducing adenoma miss rates and thus increase adenoma detection. (85% agreement)

1.2 Use of CADe is likely to increase health care costs by detecting more adenomas in the short-term.  (70% agreement)

1.3 In the long-term, this cost increment could be balanced by savings in costs related to cancer treatment (surgery, chemotherapy, palliative care) due to CADe-related cancer prevention. (85% agreement)

1.4 Health insurance bodies may be inclined to introduce reimbursement if reliable data exist for the overall cost-effectiveness of CADe. (100% agreement)

***2. Computer-aided diagnosis (CADx)***

Computer-aided diagnosis (CADx) for diminutive polyps (<=5mm) is likely to reduce health care costs by reducing polypectomies, or pathological examinations, or both. Health insurance bodies should consider introducing reimbursement if reliable accuracy and cost-effectiveness data exist for CADx.  (85% agreement)

***3. CADe and CADx***

In the short-term, combined use of CADx may be a measure to mitigate the increased cost associated with the sole use of CADe. (85% agreement)

***4. Promotion of research***

We recommend that a greater variety of high-quality cost-effectiveness research should be undertaken to understand whether AI-implementation benefits populations and societies in different health care systems. (100% agreement)
