# Supplementary material for: Benefits and challenges in implementation of artificial intelligence in colonoscopy: World Endoscopy Organization position statement
Source: Dig Endosc. 2023 Mar 13;35(4):422–9. doi: 10.1111/den.14531 (PMC12136278; doi:10.1111/den.14531)
Supplement: Supplementary file 4 — Appendix S4. Results of the third voting. [file DEN-35-422-s002.docx]

**Supplementary material 4: Results of the third voting**

***1. Computer-aided detection (CADe)***

1.1 CADe for colorectal polyps is likely to improve colonoscopy effectiveness by reducing adenoma miss rates and thus increase adenoma detection. (100% agreement)

1.2 In the short-term, use of CADe is likely to increase health care costs by detecting more adenomas. (80% agreement)

1.3 In the long-term, the increased cost by CADe could be balanced by savings in costs related to cancer treatment (surgery, chemotherapy, palliative care) due to CADe-related cancer prevention. (80% agreement)

1.4 Health care delivery systems and authorities should evaluate the cost effectiveness of CADe to support its use in clinical practice. (100% agreement)

***2. Computer-aided diagnosis (CADx)***

2.1 CADx for diminutive polyps (<=5mm), when it has sufficient accuracy, is expected to reduce health care costs by reducing polypectomies, pathological examinations, or both. (93% agreement)

2.2 Health care delivery systems and authorities should evaluate the cost effectiveness of CADx to support its use in clinical practice. (93% agreement)

***3. CADe and CADx***

In the short-term, combined use of CADx may be a measure to mitigate the increased cost associated with the sole use of CADe. (73% agreement)

***4. Promotion of research***

We recommend that a broad range of high-quality cost-effectiveness research should be undertaken to understand whether AI-implementation benefits populations and societies in different health care systems. (100% agreement)
